# Dynamic Changes in Adiponectin and Resistin Drive Remission of Cardiometabolic Risk Biomarkers in Individuals with Obesity Following Bariatric Surgery

**DOI:** 10.3390/ph17020215

**Published:** 2024-02-07

**Authors:** Amanda Machado Fiorotti, Amanda Cristina Araújo Gomes, Amanda Motta Bortoli, Beatriz Bobbio de Brito, Karolini Zuqui Nunes, Fabiano Kenji Haraguchi, Andressa Bolsoni-Lopes

**Affiliations:** Postgraduate Program in Nutrition and Health, Health Sciences Center, Federal University of Espirito Santo, Vitoria 29047-105, Brazilamandacristina.ag94@gmail.com (A.C.A.G.); amandamb15@gmail.com (A.M.B.); beatrizbobbio2016@gmail.com (B.B.d.B.); karol-zuqui@hotmail.com (K.Z.N.); fabianokenji@gmail.com (F.K.H.)

**Keywords:** bariatric surgery, obesity management, abdominal adiposity, cardiometabolic risk factors, adiponectin, resistin

## Abstract

The remission of obesity-related diseases following bariatric surgery appears to result from the reorganization of metabolic and hormonal pathways involving adipokines. This study aimed to investigate the relationship between changes in body adiposity and serum adipokine levels, as well as the association between variations in adiponectin or resistin levels and cardiometabolic risk blood biomarkers before and after Roux-en-Y gastric bypass. A longitudinal and prospective study was conducted with bariatric surgery patients. Anthropometric, body composition and blood biochemical parameters were measured before and at 2 and 6 months post-surgery. The data were analyzed using ANOVA, Pearson or Spearman correlation, and simple linear regression with a significance level of *p* < 0.05. Among 36 mostly female patients aged 30 to 39 years, significant reductions in body weight (−26.8%), fat mass (−50%), waist circumference (−18%) and waist-to-height ratio (−22%) were observed post-surgery. Serum adiponectin levels increased (+107%), while resistin (−12.2%), TNF-α (−35%), and PAI-1 (−11.1%) decreased. Glucose, insulin, CRP, cholesterol, LDL-c, triglycerides, and vitamin D also decreased. Waist circumference variation showed a positive correlation with PAI-1 and TNF-α and a negative correlation with adiponectin. The total fat mass showed a positive correlation with PAI-1. Adiponectin variation correlated negatively with glucose, resistin, and CRP but positively with HDL-c. Resistin showed a positive correlation with insulin and CRP. In conclusion, 6 months post-bariatric surgery, reducing abdominal adiposity had a more significant impact on serum adipokine levels than total fat mass. Adiponectin increase and resistin decrease acted as endocrine mediators driving the remission of cardiometabolic risk biomarkers in individuals with obesity following Roux-en-Y gastric bypass.

## 1. Introduction

Obesity is a chronic, progressive, and recurrent disease that affects over 650 million people worldwide [1]. In Brazil, data from the Ministry of Health [2] indicate that currently, 60.3% of adults are overweight, which is equivalent to 96 million people, with approximately 22% having obesity.

Obesity is characterized by an excessive increase in white adipose tissue mass, along with the dysregulation of its function as a metabolic and endocrine organ. This imbalance causes low-intensity chronic inflammation and disrupts energy homeostasis, contributing to the development of chronic cardiovascular, metabolic, and immunological diseases [3,4,5,6]. Visceral adipose tissue hypertrophy, combined with increased macrophage infiltration, results in unbalanced adipokine secretion, culminating in a greater release of pro-inflammatory and oxidative molecules and a reduction in anti-inflammatory molecules [4,7,8,9].

Biological markers, or biomarkers, have been utilized to predict the risk or progression of cardiovascular and metabolic diseases, providing crucial prognostic insights into the disease course and/or therapeutic interventions [10,11]. Alongside conventional markers like blood glucose, lipid profile, C-reactive protein (CRP), and troponin I, recent scientific literature has underscored new markers, produced partially or exclusively in adipose tissue, including adiponectin, resistin, TNF-α, and plasminogen activator inhibitor-1 (PAI-1) [7,11,12].

Adiponectin is an adipokine produced in white adipocytes that is inversely associated with obesity [4]. This adipokine plays a beneficial role in regulating metabolism, glycemic control, and anti-inflammatory responses [7,11,13]. On the other hand, resistin, an adipokine produced in the vascular stroma of human white adipose tissue, is known for its role in promoting insulin resistance and inflammation [14] with associated elevated concentrations of type 2 diabetes mellitus (DM2) and obesity [4,7].

Seeking control of severe obesity refractory to clinical treatments, bariatric surgery, specifically, Roux-en-Y gastric bypass, is an effective therapeutic strategy for weight loss and the remission of metabolic syndrome, which is a surgical technique that involves a combination of anatomical reductions in stomach and intestinal disabsorption [6,15,16].

The mechanisms involved in this process of comorbidity remission remain under investigation, as the simple restriction of food intake or a combination of restriction and malabsorption induced by the bypass is not enough to explain the effects of such complexity [6,17].

More recent studies demonstrate that this surgery is capable of promoting a reorganization of metabolic and hormonal pathways involving, among others, adipokines engagement, such as leptin, adiponectin, and orexin-A [17,18,19].

Regardless, it is still necessary to expand our understanding of the association between rapid changes in body composition and changes in the concentration of circulating adipokines, as well as the contribution of adipokines to the metabolic rearrangement experienced by patients after short-term bariatric surgery, which favors energy homeostasis and reduced cardiometabolic diseases. Our hypothesis posits that the rapid shifts in body composition observed post-Roux-en-Y gastric bypass are linked to substantial changes in anti- and pro-inflammatory adipokines, with a particular emphasis on adiponectin and resistin. These adipokines potentially influence the remission of cardiometabolic risk factors.

Therefore, this study aimed to investigate the relationship between changes in body adiposity and serum adipokine levels, as well as the association between variations in adiponectin or resistin levels and blood biomarkers of cardiometabolic risk before and after Roux-en-Y gastric bypass.

This study provides relevant and new information regarding the association between abdominal adiposity variation and changes in the serum levels of adipokines promoted by short-term bariatric surgery. They also highlight that the reduction in cardiometabolic risk biomarkers after Roux-en-Y gastric bypass involves endocrine regulation mechanisms, with adiponectin and resistin acting as mediators of this process. The study emphasizes the use of biochemical and anthropometric data, with an emphasis on waist circumference, as alternatives to monitor patients’ health and manage metabolic risk related to obesity both before and after bariatric surgery.

## 2. Results

This study involved the participation of 36 volunteer patients, comprising 32 females (89%) and 4 males (11%). Approximately 72.2% of the volunteers were married, 61.1% had between 6 and 12 years of education, and 41.7% were between 30 and 39 years old, as shown in Table 1.

According to Table 2, the highest mean values for body weight, fat mass, waist circumference and waist-to-height ratio among the considered time points were observed preoperatively, while the lowest means for these parameters were observed six months postoperatively.

When evaluating the Body Mass Index (BMI) in the preoperative period, 75% of the sample was classified as Class III obesity; however, six months after surgery, only 13.3% of the sample fell into this classification. Among the patients, 63.9% lost between 10.1 kg and 20 kg of fat in the first two months; additionally, 56.6% of participants lost between 20.1 kg and 30 kg of fat six months after surgery (Table 2).

After quantifying the blood levels of adipokines and other biological markers linked to cardiometabolic diseases, it was statistically observed that the highest means are in the preoperative period for resistin, PAI-1, TNF-α, glucose, insulin, CRP, total cholesterol, LDL, triglycerides, and vitamin D compared to the postoperative data at two and six months. On the other hand, in the six-month postoperative period, the highest means were obtained for adiponectin and HDL cholesterol compared to the preoperative period. Regarding adipokines, it was possible to observe that six months after gastric bypass, there was a 107% increase in circulating levels of adiponectin, as well as a reduction of 12.2% in resistin, 11.1% in PAI-1, and 34.9% in TNF-α. No statistical differences were identified between the investigated periods for troponin I, as shown in Table 3.

Aiming to understand the relationship between body adiposity and adipokine serum levels, cross-data tests were conducted specifically to identify how changes in variables are related over time. In these tests, the delta was used, which is obtained by calculating the variation between the values recorded six months postoperatively and subtracting the values recorded preoperatively for each parameter.

In Table 4, a significant positive correlation can be observed between the variation in total fat mass and PAI-1. Furthermore, a significant positive correlation can be observed between the variation in waist circumference and PAI-1 as well as TNF-α, in addition to a negative correlation between the variation in waist circumference and adiponectin. Resistin levels were not related to changes in total fat mass or waist circumference.

Adding to the previous results, the simple linear regression test statistically confirmed the existence of an inverse association between the variation in waist circumference and adiponectin (R^2^ = 0.16; *p* ≤ 0.03); reducing one unit of waist circumference (cm) increased serum adiponectin by 0.38 µg/mL. For the other parameters observed in the correlation analysis, it was not possible to apply the linear regression test as they did not meet the prerequisites required for such analysis.

**Figure 1:**
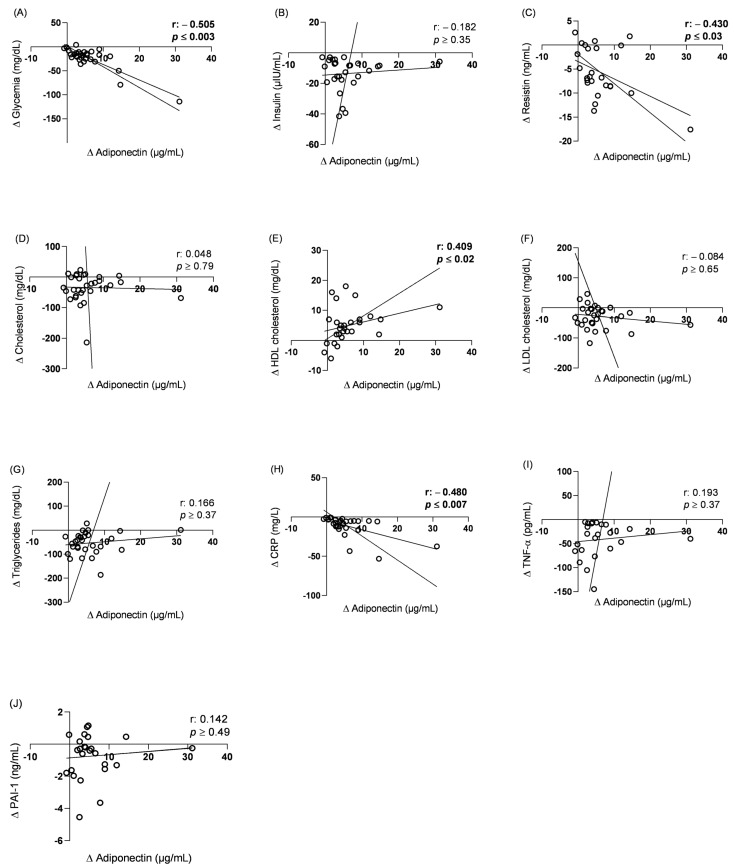
Correlation between serum adiponectin levels variation and cardiometabolic risk biomarker variation in patients preoperatively and six months after bariatric surgery (*n* = 24). (**A**) Glycemia; (**B**) Insulin; (**C**) resistin; (**D**) Total cholesterol; (**E**) HDL cholesterol; (**F**) LDL cholesterol; (**G**) Triglycerides; (**H**) CRP; (**I**) TNF-α; (**J**) PAI-1. r: Pearson’s correlation coefficient or Spearman’s correlation coefficient when normality was rejected in the Shapiro–Wilk test; *p* < 0.05. (Δ) Difference between the levels of biological analytes in patients’ serum six months after surgery and the preoperative concentration; (CRP) C-Reactive Protein; (TNF-α) Tumor necrosis factor alpha; (LDL) Low-density lipoprotein; (HDL) High-density lipoprotein; PAI-1: Plasminogen activator inhibitor-1.

Given the importance of adiponectin as a signaling and regulatory molecule of energy metabolism and inflammation in different body tissues, this research investigated the relationship between adiponectin level variations and biomarkers of cardiometabolic risk variations before and after bariatric surgery. The results revealed a significant negative correlation with glycemia, resistin and CRP, and a significant positive correlation with HDL, as shown in Figure 1.

After identifying a significant correlation between the variation in adiponectin levels and certain cardiometabolic risk biomarkers, simple linear regression analysis was applied. This revealed that an increase in one level of adiponectin unit (µg/mL) promoted a reduction of 3.22 mg/dL of glycemia (R^2^ = 0.720; *p* ≤ 0.0001), 0.36 ng/mL of resistin (R^2^ = 0.174; *p* ≤ 0.02) and 1.17 mg/L of CRP (R^2^ = 0.303; *p* ≤ 0.009). However, in this statistical test, adiponectin did not prove to be a significant predictor of variations in HDL (R^2^ = 0.065; *p* ≥ 0.09).

**Figure 2:**
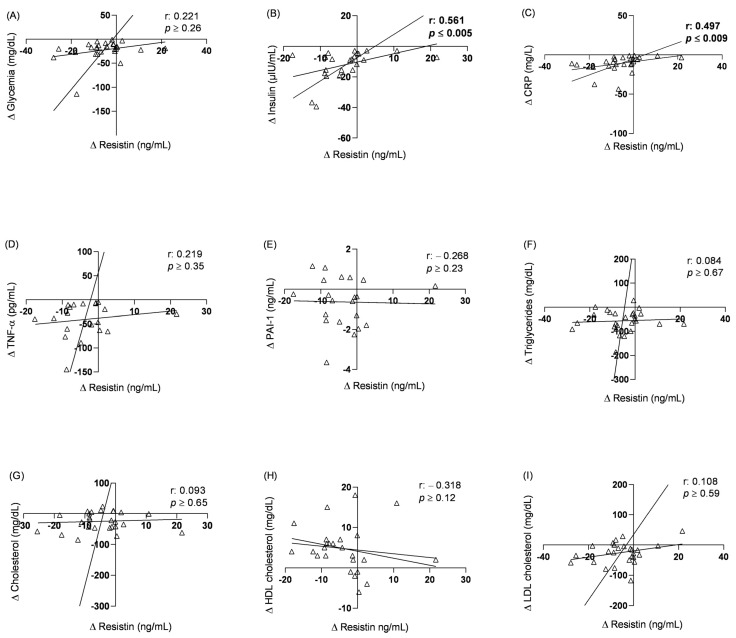
Correlation between serum resistin levels variation and cardiometabolic risk biomarker variation in patients preoperatively and six months after bariatric surgery (*n* = 24). (**A**) Glycemia; (**B**) Insulin; (**C**) CRP; (**D**) TNF-α; (**E**) PAI-1; (**F**) Triglycerides; (**G**) Total cholesterol; (**H**) HDL cholesterol; (**I**) LDL cholesterol. r: Pearson’s correlation coefficient or Spearman’s correlation coefficient when normality was rejected in the Shapiro–Wilk test; *p* < 0.05. (Δ) Difference between the concentration of biological analytes in patients’ serum six months after surgery and the preoperative concentration; (CRP) C-Reactive Protein; (TNF-α) Tumor necrosis factor alpha; (LDL) Low-density lipoprotein; (HDL) High-density lipoprotein; PAI-1: Plasminogen activator inhibitor-1.

Similarly, we sought to investigate resistin, a molecule produced in the vascular stroma of human adipose tissue, which is commonly associated with increased inflammation and worsened insulin signaling. As visualized in Figure 2, the data revealed a significant positive correlation between resistin and insulinemia and CRP. Furthermore, through simple linear regression analysis, it was identified that a reduction in one unit in resistin levels (ng/mL) resulted in a reduction of 0.54 μIU/mL of insulin (R^2^ = 0.203; *p* ≤ 0.030).

## 3. Discussion

Bariatric surgery represents a highly effective and durable treatment for refractory severe obesity, offering benefits beyond weight loss. In many cases, patients experience the remission of diseases and can discontinue the use of antihypertensives and hypoglycemic agents in a period of less than six months [20,21,22,23]. Among them, Roux-en-Y gastric bypass has proven to be highly effective in the remission of metabolic syndrome through various mechanisms still under investigation [6,15,16,24].

In the present study, we have unveiled new data to expand the understanding of such phenomena. Notably, in the short-term postoperative bypass, we observed that positive changes in circulating adipokine levels were mainly related to the abdominal adiposity/waist circumference reduction but not necessarily to total fat mass. We also demonstrated that the increase in serum adiponectin levels, along with the reduction in resistin, appear to be endocrine mechanisms that mediate the reduction in the risk of obesity-associated diseases.

In this investigation conducted in a public hospital in Brazil, the majority of individuals undergoing bariatric surgery are female, aged between 30 and 59, married, have 6 to 12 years of education and have lower incomes. The research indicates that, despite a similarity in the obesity percentage between men and women in Brazil, a significant proportion of women opt for bariatric surgery and pursue the procedure at progressively younger ages [2,25].

As expected, the surgery resulted in a 26% reduction in patient body weight, in addition to a reduction in total fat mass and waist circumference. According to the International Federation for the Surgery of Obesity and Metabolic Disorders (2018), the average weight loss in one year after surgery is approximately 28.9% [26].

Furthermore, within the period of up to six months post-bariatric surgery, there was a significant change in the serum levels of molecules that are classically described in the literature as biomarkers for cardiometabolic diseases [10,11], including the reduction in inflammatory factors (CRPs), blood glucose and insulin, as well as triglycerides and total cholesterol.

The hormones produced by white adipose tissue, known as adipokines, were also modified, with an increase in adiponectin levels and a reduction in resistin, PAI-1, and TNF-α. Interestingly, our results indicate that the decrease in waist circumference played a more significant role in the remodeling of circulating adipokine levels than the reduction in total fat mass. This leads to the consideration that the reduction in abdominal adiposity favored the physiological readjustment of adipokine production.

Waist circumference is a simple method used to assess abdominal adiposity, considered a good indicator of central obesity and a predictive parameter of risk for cardiovascular diseases. It is associated with a 60–70% higher risk of primary hypertension and all-cause cardiovascular mortality, with or without BMI adjustment. This association is due to the significant activation of the renin–angiotensin system, endothelial dysfunction, and sympathetic activation [27,28]. It is suggested that waist circumference and the waist-to-height ratio (WHtR) have an even stronger correlation with the development of type 2 diabetes than BMI [29,30,31].

Reinforcing these arguments, as described by Ashwell and Gibson, WHtR as a biomarker of early health risk, values above 0.5 indicate ‘increased risk’, while values above 0.6 indicate ‘very high risk’ [29]. In our study, six months after surgery, a significant reduction in WHtR was observed, transitioning from average values indicating ‘very high risk’ to ‘high risk’.

The International Atherosclerosis Society and International Chair on Cardiometabolic Risk Working Group on Visceral Obesity consensus (2020) [28] recommend that waist circumference measurement, which has been neglected, be reintegrated into clinical practice, as it is a critical factor that can be used to infer the reduced risk of cardiovascular diseases after adopting treatment strategies. In this context, this research emphasizes the importance of interpreting the results obtained from waist circumference measurement for the stratification of health risks related to obesity. Together with BMI, it can monitor the effectiveness of bariatric surgery in reversing metabolic syndrome.

Moreover, white adipose tissue distribution plays a crucial role in regard to obesity-associated comorbidities. Metabolic syndrome is most frequently observed in individuals with a large accumulation of visceral abdominal fat, which has a high metabolic activity and constantly releases free fatty acids into portal circulation. As a result, hypertrophied visceral adipose tissue shows notable differences in adipokine release profile, including an increase in inflammatory cytokines such as TNF-α and macrophage chemoattractants (MCP-1), resistin and PAI-1, along with a reduction in adiponectin [11,32,33].

Adiponectin is a hormone derived from white adipocytes, known as an important biomarker, which regulates body energy homeostasis, metabolism and inflammation. Its reduction occurs in inflammatory conditions and obesity [7,11,13,33]. Reduced adiponectin secretion precedes insulin resistance, but increases reverse this situation [11,13,33]. Hypoadiponectinemia is also a risk indicator for coronary artery disease, high blood pressure, and atherosclerosis. Its cardioprotective effects are related to increased nitric oxide production due to eNOS activation and the prevention of endothelial apoptosis [33,34,35]. The anti-inflammatory effects of adiponectin include the suppressed production of pro-inflammatory cytokines, such as TNF-α, IL-6, and CRP, in addition to the modulation of IL-10 expression in monocytes and macrophages [11,13,33].

In this research, it was observed that the increase in the blood level of adiponectin proved to be a strong predictor of the reduction in cardiometabolic risk after bariatric surgery. This is due to its association with a decrease in blood glucose, resistin and CRP, along with the increase in HDL cholesterol. These findings align with previously published studies [17] and contribute to the scientific literature, providing a basis for understanding endocrine/paracrine/autocrine mechanisms through which mixed disabsorptive bariatric surgeries promote the reversal of metabolic syndrome.

Resistin is an adipokine produced in the vascular stroma of human white adipose tissue and has been described as a biological marker for chronic diseases, playing a crucial role in the initiation and progression of obesity, insulin resistance, cardiovascular diseases and cancer through mechanisms that involve the strong activation of inflammatory pathways dependent on Toll-like receptor 4 [4,14]. In this study, a decrease in circulating levels of resistin was observed in the first two months after surgery. In addition, the variation in resistin positively correlated with the reduction in insulin and CRP levels. These findings suggest that the decrease in resistin is also one of the mechanisms that influences the metabolic improvement of patients after Roux-en-Y bypass. Interestingly, reduced resistin levels occurred independently of changes in total fat mass or waist circumference, although it showed a correlation with increased adiponectin.

PAI-1 is a molecule often found at elevated levels in the blood of individuals with obesity and type 2 diabetes [1,36]. It is produced by endothelial cells, liver, and adipose tissue, and plays a role in various pathophysiological processes such as the regulation of fibrinolysis, inflammatory responses, and the negative regulation of glucose and lipid metabolism [11,36]. Specifically, zzz-derived PAI-1 is predominantly produced in visceral fat, where it has detrimental effects on metabolism and vascular biology [37]. Elevated plasma levels of PAI-1 have been identified as an independent indicator of the risk of cardiovascular diseases in patients who have experienced myocardial infarction [11,38].

Inflammation is a common link between obesity and cardiometabolic diseases, and TNF-α is one of the main adipokines involved in the inflammatory processes that occur in the context of obesity, mediated by NF-κB, JNK, and NLRP3 inflammasomes [9]. Dysfunctional adipocytes and infiltrated M1 macrophages in adipose tissue release TNF-α, which has atherogenic effects and promotes insulin resistance and dyslipidemia [4]. In general, it is established that visceral obesity is more strongly associated with the release of TNF-α than subcutaneous fat [9].

Although the present study has focused on adiponectin and resistin, the influence and relevance of other adipokines, such as leptin, orexin A, IL-6, or other molecules, should not be forgotten, given the complexity and breadth of the effects promoted by bariatric surgery in the human body [17,18,39,40,41,42].

Despite all the benefits promoted by bariatrics described so far, this study identified a decrease in vitamin D in the patients’ blood after surgery. Vitamin D deficiency is often associated with bone diseases; however, this vitamin has also been recently associated with the greater prevalence and severity of non-skeletal diseases, including obesity, cardiovascular diseases, DM2 and hypertension [11,43], characterizing it as a potential prognostic biomarker for chronic diseases [11]. It is worth mentioning that the Roux-en-Y gastric bypass technique presents malabsorptive characteristics that lead to nutritional deficiencies, including vitamin D, which occur due to a delayed mixing of nutrients with bile acids and pancreatic enzymes close to the jejunum and ilium [44,45].

In such a manner, the present study provided relevant information regarding the notable modification of blood levels of adipokines and their contribution to reducing cardiometabolic risk biomarkers in individuals with obesity undergoing short-term bariatric surgery. The results indicate significant metabolic improvements in patients, and such events seem to occur, at least in part, due to the increase in adiponectin and reduction in resistin resulting from the decrease in abdominal adiposity.

The evidence gathered reaffirms the understanding that the remission of diseases associated with obesity after bariatric surgery is not only due to weight loss or poor food absorption but also to the regulation of the production of metabolic and inflammatory hormonal mediators by visceral adipose tissue, including adipokines.

It is noteworthy that the biochemical assessment of adipokines, especially adiponectin and resistin, can provide relevant information to the monitoring and prognosis of people undergoing treatment for obesity. The importance of measuring anthropometric data is also emphasized, particularly routine waist circumference measurements, as they are a simple and very low-cost method that provide health professionals with a powerful tool to manage the cardiometabolic risk associated with obesity before and after bariatric surgery.

The limitations of this study are associated with the challenges inherent to a longitudinal study, such as participant attrition, cancellations of elective surgeries, rescheduling of appointments, and operational constraints in the clinical analysis laboratory. These factors, encountered throughout the study, contributed to a reduction in the sample size. Another potential limitation of the study is the random discrepancy in the sample composition between men and women, hindering the generalization of findings to both sexes.

## 4. Materials and Methods

A prospective longitudinal study was conducted at the ambulatory clinic of a university hospital in the city of Vitória, ES, Brazil, involving patients enrolled in the Bariatric and Metabolic Surgery Program. Data collection took place from July 2022 to May 2023. Patients were recruited approximately one month before surgery and invited to participate in pre-scheduled appointments.

The inclusion criteria encompassed individuals aged 18 years or older, with a BMI between 35 and 45 kg/m^2^, who underwent surgery using the Roux-en-Y bypass method [25,42,46], in accordance with the indication criteria outlined in the legislation of the Ministry of Health of Brazil [47], and expressed willingness to participate by signing the Informed Consent Form (ICF).

The exclusion criteria comprised individuals with a history of prior bariatric surgery, use of anti-obesity drugs, pregnant individuals, those with intellectual and/or communication and/or mobility limitations, uncontrolled psychiatric disorders, use of alcohol, tobacco, or illicit drugs, severe and decompensated cardiopulmonary diseases, portal hypertension with esophagogastric varices, immune or inflammatory diseases of the upper digestive tract, cancer, and infectious diseases [25,42,47]. Additionally, those with a pacemaker or any metallic structures were contraindicated for electrical bioimpedance [48] (as per European Society for Clinical Nutrition and Metabolism (ESPEN) recommendations). The discontinuity criteria involved non-completion of data collection protocol steps due to participant request or abandonment.

**Figure 3:**
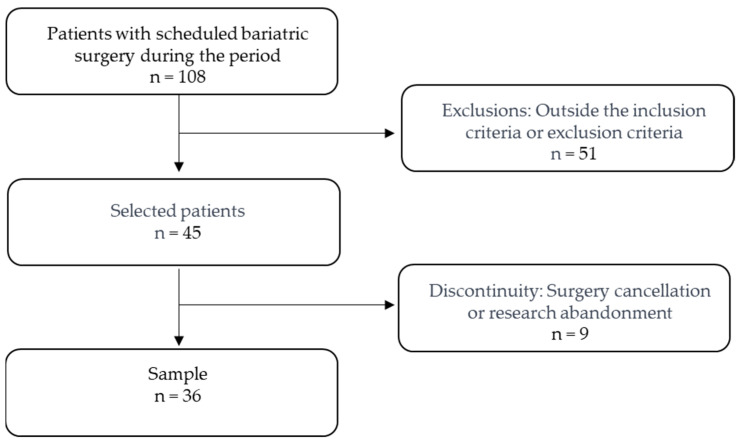
Sample selection process.

As for convenience sampling, complying with the criteria described, forty-five patients agreed to participate in the research, but seven withdrew from the study for personal reasons, and two did not undergo surgery, resulting in a final sample of thirty-six patients, as shown in Figure 3.

The Roux-en-Y gastric bypass procedure was standardized with a 40 cm biliopancreatic limb length and 150 cm alimentary limb length [25,42,46].

Data collection was carried out by researchers who were previously trained and qualified. The patients were assessed individually and privately at three consecutive time points: preoperatively, two months post-surgery, and six months post-surgery. The data collection process began with the application of a sociodemographic characterization instrument, which included information on gender, marital status, age group, and education. Following that, participants underwent the measurement of anthropometric data and bioimpedance testing (Inbody^®^ 270, Republic of Korea). Finally, patients were led to the clinical analysis laboratory, responsible for collecting the blood sample [49,50].

Blood collection was carried out after fasting for 10 to 12 h in the Hospital’s Clinical Analysis Laboratory Unit, in accordance with standardized operating procedures. Greiner bio-one^®^ 21 G needles and collection tubes without heparin, with 5 mL capacity, were used. Samples were placed on ice and centrifuged (2500 gL, 12 min at 4 °C) to obtain the serum, which was stored in a freezer at −80 °C for later analysis.

Human serum samples were used to evaluate glucose, CRP, troponin I, LDL cholesterol (LDL-c), HDL cholesterol (HDL-c), total cholesterol, and triglycerides, which were quantified using colorimetric enzymatic methods using commercial kits and automatic analyzer model CMD801 (Wiener^®^, Santa Fé, Argentina).

Concentrations of adipokines, hormones and cytokines in human serum were determined using immunoenzymatic assay kits—ELISA (Enzyme Linked Immuno Sorbent Assay). In order to determine adiponectin and resistin, SIGMA ELISA kits for humans were used (Sigma-Aldrich, Saint Louis, MO, USA; catalog numbers RAB0005 and RAB0419, respectively). To determine PAI-1, Vitamin D, TNF-α and Insulin, Elabscience^®^ ELISA kits for humans were used (Elabscience^®^ Biotechnology, Houston, TX, USA; catalog numbers E-EL-H2104, E-EL-0012, E-EL-H0109 and E-EL-H2665, respectively). The test was carried out according to the protocol provided by the companies producing the kits.

Data analysis was performed using the Statistical Package for the Social Sciences (SPSS) Window version 24 and GraphPad Prism version 8.0.2 software (GraphPad Software, Inc., San Diego, CA, USA). This consisted of a descriptive analysis, as expressed by their absolute and relative frequencies. The distribution of metric variables was evaluated by determining the mean and standard deviation. Data were analyzed using the one-way analysis of variance (ANOVA) test, followed by the Tukey or Bonferroni or Kruskal–Wallis post-test, when normality was rejected in the Shapiro–Wilk test. For data crossing, Pearson or Spearman correlation was applied when normality was rejected in the Shapiro–Wilk test. In cases of significant correlation, a simple linear regression model was applied to the variables that met at least two of the three criteria: normality of residuals, homoscedasticity, and outlier residuals. A significance level of *p* ≤ 0.05 was adopted.

National and international ethics standards for research involving human beings were met. The project was reviewed and approved by the Human Research Ethics Committee of Cassiano Antonio Morais Hospital in Vitória, under protocol number 5.517.685, dated July 2022.

## 5. Conclusions

Over six months of postoperative Roux-en-Y gastric bypass, dynamic changes were observed in the serum levels of adipokines, with an increase in adiponectin and a reduction in resistin, PAI-1 and TNF-α. These changes were mainly associated with reduced abdominal adiposity but not necessarily reduced total fat mass.

The increase in circulating adiponectin levels and the decrease in resistin significantly contributed to the remission of cardiovascular and metabolic risk biomarkers, including their association with the regulation of blood glucose, lipids, and inflammatory cytokines. These findings substantiate the concept that the metabolic improvement observed in patients after Roux-en-Y gastric bypass involves endocrine regulation mechanisms, with adipokines, particularly adiponectin and resistin, acting as mediators of this process.

## Figures and Tables

**Table 1:** Sociodemographic characterization of patients undergoing bariatric surgery (*n* = 36).

Variable	*n*	%
	**36**	**100**
Gender		
Female	32	89
Male	4	11
Marital status		
Married	26	72.2
Non married	10	27.8
Level of education		
<6 Years	6	16.7
6–12 Years	22	61.1
>12 Years	8	22.2
Age group		
20–29 years	2	5.6
30–39 years	15	41.7
40–49 years	11	30.6
50–59 years	4	11.1
≥60 years	4	11.1

*n* = number of patients; % = patients’ percentages.

**Table 2:** Anthropometric characteristics and body composition of patients preoperatively, two months and six months after bariatric surgery (*n* = 30–36).

		**Preoperative**		**After Surgery** **(2 Months)**		**After Surgery** **(6 Months)**		
		**Mean ± SD**		**Mean ± SD**		**Mean ± SD**		***p* Value**
	Body weight (kg)	119.5 ^a^ ± 22.0		98.8 ^b^ ± 16.4		87.0 ^c^ ± 17.9		**<0.001**
Anthropometric Data	Fat mass (kg)	60.2 ^a^ ± 14.2		44.7 ^b^ ± 12.0		31.9 ^c^ ± 12.9		**<0.001**
	Waist circumference (cm)	123 ^a^ ± 15.7		110 ^b^ ± 14.8		101 ^c^ ± 15.8		**<0.001**
	Waist-to-height ratio (cm/cm)	0.75 ^a^ ± 0.09		0.67 ^b^ ± 0.09		0.59 ^c^ ± 0.07		**<0.001**
		**Preoperative**		**After Surgery** **(2 Months)**		**After Surgery** **(6 Months)**		
		***n***	**%**	***n***	**%**	***n***	**%**	
BMI	Eutrophic (18.5 to 24.9 kg/m^2^)	0	0	0	0	2	6.7	
	Overweight (25 to 29.9 kg/m^2^)	0	0	1	2.8	9	30	
	Class I obesity (30 to 34.9 kg/m^2^)	0	0	17	47.2	12	40	
	Class II obesity (35 to 39.9 kg/m^2^)	9	25	10	27.8	3	10	
	Class III obesity (≥40 kg/m^2^)	27	75	8	22.2	4	13.3	
Fat mass loss	10 kg or less	-	-	7	19.4	1	3.3	
	10.1 kg to 20 kg	-	-	23	63.8	4	13.3	
	20.1 kg to 30 kg	-	-	5	13.8	17	56.6	
	30.1 kg to 40 kg	-	-	1	2.7	6	20.0	
	40.1 kg or more	-	-	0	0.0	2	6.6	

Values expressed as mean ± standard deviation of the mean (SD). One-way ANOVA followed by Bonferroni post-test; abc—different letters indicate differences between the means; significant if *p* < 0.05. Or values expressed in absolute and relative frequencies. *n* = absolute number of patients; % = percentage of patients. BMI: Body Mass Index.

**Table 3:** Serum levels of adipokines and cardiometabolic risk biomarkers in patients preoperatively, at two months, and six months after bariatric surgery (*n* = 24–34).

	Preoperative	After Surgery 2 Months	After Surgery 6 Months	*p* Value
	Mean ± SD	Mean ± SD	Mean ± SD	
Adiponectin (μg/mL)	5.4 ^a^ ± 4.1	8.5 ^b^ ± 5.5	11.3 ^c^ ±7.0	**0.002**
Resistin (ng/mL)	36.4 ^a^ ± 10.2	32.9 ^b^ ± 10.4	32.0 ^b^ ± 9.2	**0.009**
PAI-1 (ng/mL)	6.5 ^a^ ± 1.1	6.1 ^a,b^ ± 1.0	5.8 ^b^ ± 0.4	**0.04**
TNF-α (pg/mL)	87.9 ^a^ ± 47.7	60.3 ^b^ ± 36.1	57.2 ^b^ ± 27.4	**<0.001**
Glucose (mg/dL)	111.3 ^a^ ± 25.2	92.1 ^b^ ± 11.0	87.3 ^b^ ± 11.4	**<0.001**
Insulin (μIU/mL)	26.1 ^a^ ± 19.0	16.1 ^a,b^ ± 17.1	9.9 ^b^ ± 3.3	**<0.001**
CRP (mg/dL)	15.0 ^a^ ± 17.1	5.1 ^b^ ± 4.5	3.5 ^b^ ± 4.1	**<0.001**
Troponin I (ng/mL)	0.05 ^a^ ± 0.1	-	0.05 ^a^ ± 0.02	0.49
Total cholesterol (mg/dL)	183 ^a^ ± 37.2	153 ^b^ ± 37.8	151 ^b^ ± 32.2	**<0.001**
LDL (mg/dL)	114 ^a^ ± 34.7	92 ^b^ ± 26.5	81 ^b^ ± 23.8	**<0.001**
HDL (mg/dL)	43 ^a^ ± 10.1	40 ^a^ ± 9.2	47 ^a^ ± 13.3	**0.005**
Triglycerides (mg/dL)	153.9 ^a^ ± 91.0	108.5 ^a,b^ ± 47.6	88.8 ^c^ ± 41.6	**<0.001**
Vitamin D (ng/mL)	49.9 ^a^ ± 12.0	44.0 ^a,b^ ± 12.1	40.9 ^b^ ± 12.3	**<0.001**

Analysis of Variance (ANOVA); abc—different letters indicate differences between the means; *p* < 0.05. CRP: C-reactive protein; TNF-α: Tumor necrosis factor—alpha; PAI-1: Plasminogen activator inhibitor-1; LDL: low-density lipoprotein; HDL: high-density lipoprotein.

**Table 4:** Correlation between the variation in total fat mass or waist circumference with the variation in the blood levels of adipokines in patients preoperatively and six months after bariatric surgery (*n* = 24–34).

Dependent Variables	Independent Variables	r	*p* Value	95% CI	
				Lower Limit	Upper Limit
Δ Adiponectin (μg/mL)	Δ Fat mass	−0.291	0.16	−0.6291	0.1398
	Δ WC	−0.485	**0.008**	−0.7324	−0.1260
Δ Resistin (ng/mL)	Δ Fat mass	0.33	0.12	−0.1073	0.6611
	Δ WC	0.079	0.76	−0.4296	0.5506
Δ PAI-1 (ng/mL)	Δ Fat mass	0.452	**0.002**	0.05802	0.7252
	Δ WC	0.418	**0.04**	0.0052	0.7149
Δ TNF-α (pg/mL)	Δ Fat mass	−0.098	0.70	−0.5637	0.4144
	Δ WC	0.484	**0.04**	−0.0106	0.7887

r: Pearson’s correlation coefficient or Spearman’s correlation coefficient when normality was rejected in the Shapiro–Wilk test; significant if *p* < 0.05. Δ WC: Waist circumference delta; TNF-α: Tumor necrosis factor—alpha; PAI-1: Plasminogen activator inhibitor type 1; Δ: delta.

## Data Availability

Data are contained within the article.
